# Unusual presentation of hereditary neuropathy with liability to pressure palsies

**DOI:** 10.1186/1749-7221-3-2

**Published:** 2008-01-24

**Authors:** Muhammad U Farooq, Jayne HW Martin, Michael T Andary

**Affiliations:** 1 Department of Neurology and Ophthalmology, Michigan State University, East Lansing, MI, USA; 2 Department of Physical Medicine and Rehabilitation, Michigan State University, College of Osteopathic Medicine, East Lansing, MI, USA

## Abstract

**Background:**

Hereditary neuropathy with liability to pressure palsies (HNPP) is an autosomal-dominant painless peripheral neuropathy characterized by episodes of repeated focal pressure neuropathies at sites of entrapment/compression, with a considerable variability in the clinical course. Electrodiagnostic and genetic testing are important in the diagnostic evaluation of these patients.

**Case presentation:**

We report an unusual HNPP phenotype, five compression neuropathies in four nerves in a patient with bilateral hand numbness. A 42-year-old female, presented with acute bilateral paresthesias and weakness in her hands after starting yoga exercises requiring hyperextension of her hands at the wrists. Her presentation was complicated by: a) a remote history of acute onset foot drop and subsequent improvement, b) previous diagnoses of demyelinating peripheral neuropathy, possibly Charcot-Marie-Tooth disease, and c) exposure to leprosy. Electrodiagnostic testing showed 5 separate compression neuropathies in 4 nerves including: severe left and right ulnar neuropathies at the wrist, left and right median neuropathies at the wrist and left ulnar neuropathy at the elbow. There was a mild generalized, primarily demyelinating, peripheral polyneuropathy. Based on the clinical suspicion and electrodiagnostic findings, consistent with profound demyelination in areas of compression, genetic analysis was done which identified a deletion of the PMP-22 gene consistent with HNPP.

**Conclusion:**

HNPP can present with unusual phenotypes, such as 5 separate mononeuropathies, bilateral ulnar and median neuropathies at the wrists and ulnar neuropathy at the elbow with mild peripheral demyelinating polyneuropathy associated with the PMP-22 gene deletion.

## Background

Hereditary neuropathy with liability to pressure palsies (HNPP) is an autosomal-dominant, painless peripheral neuropathy characterized by episodes of repeated focal pressure neuropathies at common sites of entrapment and compression [1,2]. There is a considerable heterogeneity in phenotypes and clinical course of this disease. Therefore electrodiagnostic, histopathologic, as well as genetic testing are important in the diagnostic evaluation of HNPP [3]. Electrophysiologically, HNPP is characterized by a generalized demyelinating neuropathy, with superimposed focal entrapment neuropathies [1,4]. There are different genotypes including a 1.5 Mb deletion of locus 17p11-2 (PMP-22 gene deletion). Pathologically, tomaculae are seen on sensory as well as motor nerves [5,6]. We report a novel phenotype of HNPP with PMP-22 gene deletion.

## Case presentation

### History and course

A 42-year-old female physician presented with bilateral hand weakness, numbness, and tingling, in the right greater than left upper extremity. The sensory symptoms involved all fingers but were most prominent in the ulnar distribution. She noted bilateral hand weakness and was not able to cross her fingers. These symptoms began the day after starting yoga exercises, which required prolonged hyperextension of her hands at the wrists. Prior to this acute presentation, after repeat questioning, she acknowledged that she did have some nocturnal paresthesias that were not bothersome.

She reported a history of peroneal neuropathy at the right fibular head eight years prior to this episode, which presented with acute onset of foot drop and subsequent improvement. She has had multiple exposures to leprosy while living in India. She denied any rashes, or skin lesions. She was seen by a physician 8 years prior to this presentation and had been told she had a demyelinating neuropathy and carpal tunnel syndrome (CTS). She never underwent complete diagnostic work up, though she was treated with B12 at that time. She, being a physician, thought that she may have Charcot-Marie-Tooth disease, but this had never been diagnosed. She had no family history of a similar problem.

### Physical examination

She was a healthy female of normal physique with intact memory, attention, orientation language and visual-spatial function. Cranial nerves II-XII were intact. There was normal tone in upper and lower extremities. Motor strength was grade 5 in all the four extremities, with the exception of mild to moderate weakness of her intrinsic hand muscles (dorsal and palmar interosseous, abductor digiti minimi; right greater than left) There was mild atrophy of intrinsic hand muscles on the right side. There was no weakness of her thumb abductors, flexor digitorum superficialis and flexor digitorum profundus. The strength in her legs was normal including the peroneal musculature. There was decreased sensation to touch and pinprick in the 4^th ^and 5^th ^digits of her right hand, and lateral side of her right thigh. Deep tendon reflexes were normal with flexor plantar responses bilaterally. Cerebellar function and gait exam were normal.

### Laboratory work up

Extensive work up for neuropathy including but not limited to routine blood tests, HbA1c, erythrocyte sedimentation rate, thyroid stimulating hormone, vitamin E, vitamin B12, creatinine kinase, anti-nuclear antibodies, and rheumatoid factor was negative.

### Electrodiagnostic studies

She had two electromyographic (EMG) studies done within the interval of one week, first on the right and second on the left side (see table 1, 2, 3, 4 for details). The electrodiagnostic abnormalities were consistent with 5 different compression neuropathies in only 4 nerves:

**Table 1:** Sensory, motor nerve conduction studies and late responses (Right)

Nerve	Stimulation Site	Record	Amplitude (mV)	DL (msec)	CV (m/sec)
**Sensory nerve conduction studies**					

Median	Wrist/midpalm	D2	Absent	Absent	-
Ulnar	Wrist	D5	Absent	Absent	-
Sural	Calf	Ankle	18	4.1	14

**Motor nerve conduction studies**					

Ulnar	Wrist	Thenar	1.1	4.5	
Ulnar	Wrist	ADM	3.5	5.0	
Ulnar	Below Elbow	ADM	3.5	8.0	51
Ulnar	Above Elbow	ADM	3.5	10.3	53
Ulnar	Wrist	FDI	2.3	6.1	
Ulnar	Below Elbow	FDI	1.8	9.2	41
Ulnar	Above Elbow	FDI	1.7	12.1	48
Ulnar	Wrist	Interosseous	0.3	7.0	
Median	Wrist	Lumbrical	0.4	13.9	
Median	Wrist	APB	4	14.1	
Median	Elbow	APB	4	18.9	46
Tibial	Ankle	AH	14	5.7	
Tibial	Knee	AH	10	13.4	46
Peroneal	Ankle	EDB	5.9	6.3	
Peroneal	Below knee	EDB	5.5	12.5	43
Peroneal	Above knee	EDB	5.4	14.2	54

**Late responses**					

Ulnar F wave	Wrist	ADM		34.3	
Tibial F-wave	Ankle	AH		51.7	
Peroneal F-wave	Ankle	EDB		49.7	
Tibial H reflex	Knee	Soleus	4	28.6	

**Table 2:** Needle Electromyography (Right)

Muscle	IA	Fibrillation	Effort	Recruitment	Polyphasia
Deltoid	N	0	N	N	N
Biceps	N	0	N	N	N
Pronator Teres	N	0	N	N	N
EDC	N	0	N	N	N
Triceps	N	0	N	N	N
FCU	N	0	N	N	N
FCR	N	0	N	N	N
APB	N	0	N	N	N
1^st ^DIM	Sustained	2+	N	Decreased	N
ADM	Sustained	3+	N	Decreased	N
Anterior Tibialis	N	0	N	N	N
Extensor Hallucis	N	0	N	N	N
Gastroc/Soleus	N	0	N	N	N

**Table 3:** Sensory, motor nerve conduction studies and late responses (Left)

Nerve	Stimulation site	Record	Amplitude (mV)	DL (msec)	CV (m/sec)
**Sensory nerve conduction studies**					

Median	Wrist/palm	D2	Absent	Absent	-
Ulnar	Wrist	D5	5	5.5	14
Radial	Wrist	D1	4	4.3	14
Sural	Calf	Ankle	16	5.0	14

**Motor nerve conduction studies**					

Ulnar	Wrist	Thenar	4.2	4.7	
Ulnar	Wrist	ADM	4.0	5.4	
Ulnar	Below Elbow	ADM	4.1	8.2	55
Ulnar	Above Elbow	ADM	4.3	11.5	34
Median	Wrist	APB	5.5	13.6	
Median	Elbow	APB	5.5	19.1	44
Tibial	Ankle	AH	15.3	5.5	
Tibial	Knee	AH	11.4	14.2	40
Peroneal	Ankle	EDB	4.8	6.5	
Peroneal	Below Knee	EDB	4.3	12.3	45
Peroneal	Above Knee	EDB	4.5	14.3	46

**Late responses**					

Tibial H reflex	Knee	Soleus	5	28.5	
Tibial F wave	Knee	AH		50	

**Table 4:** Needle Electromyography (Left)

Muscle	IA	Fibrillation	Effort	Recruitment	Polyphasia
Deltoid	N	0	N	N	N
Pronator Teres	N	0	N	N	N
EDC	N	0	N	N	N
Triceps	N	0	N	N	N
FCU	N	0	N	N	N
FCR	N	0	N	N	N
FDP D4 D5	N	0	N	N	N
APB	N	0	N	N	N
1^st ^DI Manus	N	2+	N	Decreased	Increased
Anterior Tibialis	N	0	N	N	N
Abductor Hallucis	N	0	N	N	N
Gastroc/Soleus	N	0	N	N	N

1. Left median neuropathy at the wrist

2. Right median neuropathy at the wrist

3. Right ulnar neuropathy at the wrist

4. Left ulnar neuropathy at the wrist

5. Left ulnar neuropathy at the elbow

Additionally, there was an evidence of a mild generalized primarily demyelinating peripheral polyneuropathy.

Based on the clinical suspicion and EMG findings leprosy and HNPP were considered as possible diagnoses.

### Genetic testing

The direct genetic testing for PMP-22 gene mutations was performed by PCR amplification and automated sequencing of both genomic DNA strands for all exons coding for the mature protein. The highly conserved exon-intron splice junctions between exons were also examined. Genetic analysis identified a deletion of the PMP-22 gene consistent with HNPP.

### Follow up course

The patient underwent right carpal tunnel and right Guyon canal release procedures and her symptoms improved post-operatively. She did not have any progression or new symptoms at her 6 month follow up visit at our Neurology clinic. She declined further follow up.

## Discussion

To our knowledge, this is the first case of HNPP presenting with five separate simultaneous electrodiagnostically documented compression neuropathies. These include: bilateral median and ulnar neuropathies at the wrist and ulnar neuropathy at the elbow. In this case the ulnar neuropathies were the most symptomatic. Her presentation was complicated by the confounding variables of previous diagnoses of "atypical" chronic inflammatory demyelinating polyneuropathy (CIDP) and exposure to leprosy, which could present as a demyelinating polyneuropathy.

The electrodiagnostic evidence for each of the compression neuropathies is included below.

### • Right median neuropathy at the wrist

a) prolonged absolute motor DL to APB (14.1 msec); b) prolonged absolute motor DL to lumbrical (13.9 msec); and c) absent SNAP to D2; d) comparative slowing of median motor DL to the ABP (14.1 msec) when compared to ulnar motor DL to ADM (5.0 msec); e) comparative slowing of median motor DL to the lumbrical (13.9 msec) when compared to ulnar motor DL to ADM (5.0 msec).

### • Left median neuropathy at the wrist

a) prolonged motor DL to APB (13.6 msec); b) absent SNAP to D2; c) large latency difference between median (13.6 msec) and ulnar motor DL (4.2 msec) recorded from the thenar muscles (median much slower).

### • Right ulnar neuropathy at the wrist

a) prolonged absolute motor DL to ADM (5.0 msec); b) low amplitude to ADM (3.5 mV); c) prolonged absolute motor DL to FDI (6.1 msec); d) low amplitude to FDI (2.3 mV); e) mildly prolonged ulnar absolute motor DL (4.5 msec) to thenar; f) absent SNAP to D5; g) abnormal needle EMG in the ulnar hand muscles with spared ulnar forearm muscles.

### • Left ulnar neuropathy at the wrist

a) prolonged absolute motor DL to ADM (5.4 msec); b) mildly prolonged absolute ulnar motor DL to thenar (4.2 msec); c) prolonged absolute sensory DL to D5 (5.5 msec); d) low amplitude ulnar SNAP to D5 (5 microvolts), (Note: this could also be due to ulnar neuropathy at elbow); e) abnormal needle EMG to FDI, (Note: this could also be due to ulnar neuropathy at elbow).

### • Left ulnar neuropathy at the elbow

a) slowed absolute NCV across the elbow, (34 m/sec); b) drop of NCV from the forearm (55 m/sec) compared to elbow (34 m/sec).

### • Mild generalized primarily demyelinating PPN

a) prolonged absolute radial sensory DL (4.2 msec over 10 cm); b) prolonged absolute right sural sensory DL (4.1 msec); c) slightly prolonged absolute right peroneal DL to EDB (6.3 msec); d) prolonged absolute left sural sensory DL (5.0 msec); e) slightly prolonged absolute left peroneal DL to EDB (6.5 msec).

The weakness and sensory symptoms were most consistent with bilateral ulnar neuropathies superimposed on possible bilateral CTS and peripheral polyneuropathy of unclear etiology. The polyneuropathy was essentially asymptomatic at the time of presentation but had been symptomatic in the past. The less likely diagnoses were bilateral brachial plexopathy, bilateral cervical radiculopathy or central nervous system problem.

Further history and electrodiagnostic testing, as mentioned above, were required to assist with reaching a final diagnosis. Electrodiagnostic testing confirmed the presence of a demyelinating neuropathy, in addition to an evidence of multiple compressive neuropathies. The etiology for the demyelinating peripheral polyneuropathy was

unclear, though HNPP was thought to be logical in this setting; leprosy neuropathy (Hansen's disease) or CIDP were also possible, though less logical, in this case. The previous chronic history and lack of skin rash made leprosy unlikely. The improvement in her symptoms and marked compression neuropathies made CIDP unlikely. At this point genetic testing helped to reach the final diagnosis of HNPP.

The majority of the documented cases of HNPP with ulnar nerve involvement, presented with nerve entrapment or compressive neuropathy at elbow [1,2,4,7]. There are multiple cases of HNPP presenting with CTS. However, to the best of our knowledge, there is no reported case of 5 separate electrodiagnostically documented compression neuropathies as the presentation of HNPP.

HNPP is a well documented autosomal dominant disorder characterized by recurrent pressure palsies typically precipitated by minor trauma or compression, first described by De Long in 1947[1,3,8]. In 1993 Chance et. al. showed that HNPP was due to 1.5 Mb deletion in the 17p 11-2 region spanning the gene for peripheral myelin protein 22 (PMP-22) [5]. The typical HNPP presentation may also occur with small deletions and distinct mutations rather than the 1.5 Mb deletion of the PMP22 gene [1,2,5].

HNPP typically presents with symptoms in the form of focal mononeuropathy involving median, ulnar, radial and peroneal nerves [1,3]. Atypical presentations of HNPP include acute multiple mononeuropathies in a fulminant manner, rapidly progressive peripheral nerve dysfunction, recurrent poly radiculopathies, plexopathies, acute vocal cord and hypoglossal nerve paralysis, Charcot-Marie-Tooth disease, atypical Guillain-Barre syndromes, CIDP and motor neuron disease like picture [1,9-14]. HNPP has electrophysiological findings including: evidence of focal axonal loss, diffuse sensory nerve conduction slowing, prolongation of distal motor latencies, with relatively infrequent and minor reduction of motor nerve conduction velocities [1,7,14,15]. Focal thickening of the myelin sheath, also named tomacula, are the main histopathologic characteristic of HNPP on nerve biopsy [6,15]. There are some unknown mechanisms in the pathophysiology of the disease process because mechanical factors exclusively cannot explain the recurrent nerve palsies in all cases of HNPP [9].

## Conclusion

HNPP can present with different and unusual phenotypes. Our case presented with 5 separate compression neuropathies. These include: bilateral ulnar and median neuropathies at the wrist and an ulnar neuropathy at the elbow in addition to a generalized primarily demyelinating peripheral polyneuropathy.

## Abbreviations

ADM – Abductor digiti minimi; APB – Abductor polices brevis; AH – Abductor hallucis; CV – Conduction velocity; CTS – Carpel tunnel syndrome; FDI – First dorsal interossei muscle; DL-Distal latency; D2 – 2^nd ^digit; D4 – 4^th ^digit; D5 – 5^th ^digit; EDB – Extensor digitorum brevis; EDC – Extensor digitorum communis; FCU – Flexor carpi ulnaris; FCR – Flexor carpi radialis; IA – Insertional activity; N – Normal; PPN – Peripheral polyneuropathy; SNAP – Sensory nerve action potential.

## Competing interests

The author(s) declare that they have no competing interests.

## Authors' contributions

All of the authors of this manuscript participated in the conception and design, acquisition of data, analysis and interpretation of data, drafting of the manuscript, administrative, technical, and material support.
